# Application of the Time-Domain Multichromophoric Fluorescence Resonant Energy Transfer Method in the NISE Programme

**DOI:** 10.1021/acs.jctc.4c01135

**Published:** 2024-12-24

**Authors:** Kai Zhong, Vesna Erić, Hoang Long Nguyen, Kim E. van Adrichem, Gijsbert A. H. ten Hoven, Marick Manrho, Jasper Knoester, Thomas L. C. Jansen

**Affiliations:** † Zernike Institute for Advanced Materials, University of Groningen, Nijenborgh 3, 9747 AG Groningen, The Netherlands; ‡ Faculty of Science, Leiden University, Einsteinweg 55, 2300 RA Leiden, The Netherlands; § School of Chemistry, Chemical Engineering and Biotechnology, Nanyang Technological University, 21 Nanyang Link, 637371 Singapore; ∥ Max Planck Institute for Polymer Research, Ackermannweg 10, 55128 Mainz, Germany

## Abstract

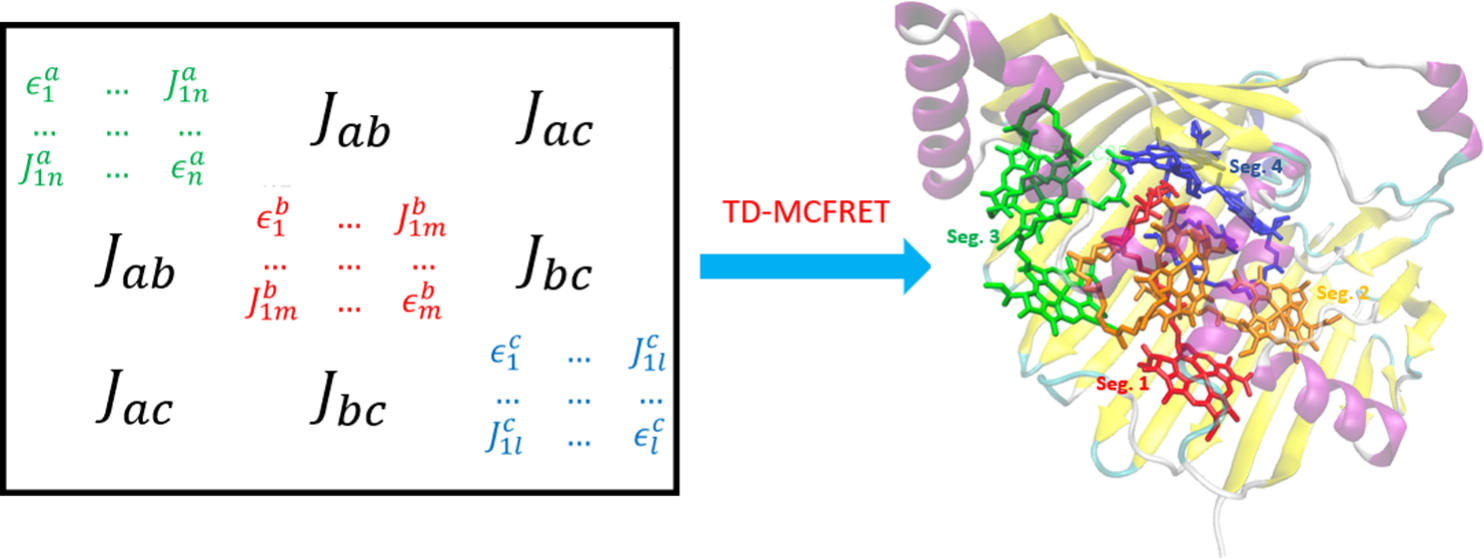

We present the implementation of the time-domain multichromophoric fluorescence resonant energy transfer (TC-MCFRET) approach in the numerical integration of the Schrödinger equation (NISE) program. This method enables the efficient simulation of incoherent energy transfer between distinct segments within large and complex molecular systems, such as photosynthetic complexes. Our approach incorporates a segmentation protocol to divide these systems into manageable components and a modified thermal correction to ensure detailed balance. The implementation allows us to calculate the energy transfer rate in the NISE program systematically and easily. To validate our method, we applied it to a range of test cases, including parallel linear aggregates and biologically relevant systems like the B850 rings from LH2 and the Fenna-Matthews-Olson complex. Our results show excellent agreement with previous studies, demonstrating the accuracy and efficiency of our TD-MCFRET method. We anticipate that this approach will be widely applicable to the calculation of energy transfer rates in other large molecular systems and will pave the way for future simulations of multidimensional electronic spectra.

## 1 Introduction

In supramolecular chromophore systems, after excitation by light, the excitation energy funnels through a network of different molecular structures.^1−3^ Transient absorption^4^ and two-dimensional electronic spectroscopy experiments are used to map the energy transfer pathways within such systems.^5,6^ The presence of many interactions in such complex systems leads to spectral congestion, requiring advanced computational models for detailed interpretation of the mentioned experiments.^7^ Such interpretation can answer key questions on the energy pathways and mechanisms utilized in biological and artificial systems for efficient energy transfer. The importance of understanding energy transfer in different materials inspired the development of several theoretical models.^8−19^ Only a few software packages are available that allow the simulation of two-dimensional electronic spectra,^20,21^ and these have a limited choice of energy transfer models available. Here, we will report on implementing the recently developed time-domain multichromophoric fluorescence resonant energy transfer (TD-MCFRET) approach^18^ in the publicly available Numerical Integration of the Schrödinger Equation (NISE) program.^20^ Our method does not rely on specific assumptions or models for the spectral density since it uses trajectories for the time-dependent Hamiltonian created from stochastic models or extracted from molecular dynamics simulations.^7^ While in the examples we will present, we will only apply simple Brownian oscillator models that can also be used in more exact approaches, the current implementation is not limited to such models, and trajectories are allowed with arbitrary bath dynamics, which even may be different for different chromophores. As such, our method provides a means for including the effects of structural and functional dynamics of molecular systems on the energy transfer process.

Natural light-harvesting systems and their synthetic analogues keep attracting notable attention due to their efficient excitation energy transfer (EET).^22−25^ The natural systems show a significant variation in pigment composition, organization, and size, depending on their environments or even light conditions.^26^ They are typically composed of tens of pigments, like in the bacterial light-harvesting complexes, Light harvesting 2 (LH2), and the Fenna–Matthews–Olson (FMO) complex. Both systems have attracted much attention because of the EET process within and between them.^27−30^ In green plants and algae, the photosynthesis supercomplex system includes the photosystem I (PSI) and II (PSII) complexes. The EET within the PSII system has been studied a lot, both theoretically and experimentally.^31−40^

Still, the complexity and size of natural systems present a substantial challenge for creating theoretical models describing energy transfer processes. A number of techniques exist to describe the EET process. The Förster resonant energy transfer theory calculates the EET rate based on the overlap between the single donor emission spectrum and single acceptor absorption spectrum and is inversely proportional to the sixth power of the donor–acceptor distance.^41,42^ In the 1990s, this theory was extended to describe EET between multichromophoric^43,44^ subsystems, denoted as the multichromophoric fluorescence resonant energy transfer (MCFRET) method, which can describe the transfer pathways between multiple coupled donors and/or acceptors. The Redfield theory^45^ and modified Redfield theory^46^ are popular methods to describe the EET process in photosynthetic systems applicable in the weak system bath coupling limit. The hierarchical equations of motion (HEOM)^14,47^ and hierarchy of pure states (HOPS)^48^ methods are formally exact methods for predicting EET. However, these methods are very demanding computationally. Here, we will focus on a recently developed trajectory-based version of TD-MCFRET^18^ and a user-friendly implementation in an existing spectral simulation program. The TD-MCFRET method can simulate the EET rate for large systems with high accuracy and computational efficiency,^18^ which is important for photosynthetic systems.

The NISE program^49,50^ is a trajectory-based program originally developed for simulating absorption and two-dimensional infrared spectra^51,52^ and later extended to other (nonlinear) electronic spectroscopies^50,53^ and two-dimensional infrared-Raman spectroscopy.^54^ The program also contains modules for studying energy transfer by solving the time-dependent Schrödinger Equation directly to obtain the population relaxation, which can describe the coherent and incoherent transfer process.^55,56^ In this paper, we report on implementing the recently developed TD-MCFRET approach^18^ and its application to relevant example systems.

In the following, we will first give a summary of the implemented method and the relevant analysis methods. Next, in the Results and Discussion section, we will demonstrate the applicability of the program by examining several examples of high relevance for electronic energy transfer. We will further discuss several implementation details and the possibilities for performing parallel computations. Finally, we will provide a conclusion and outlook.

## 2 Methods

**Figure 1:**
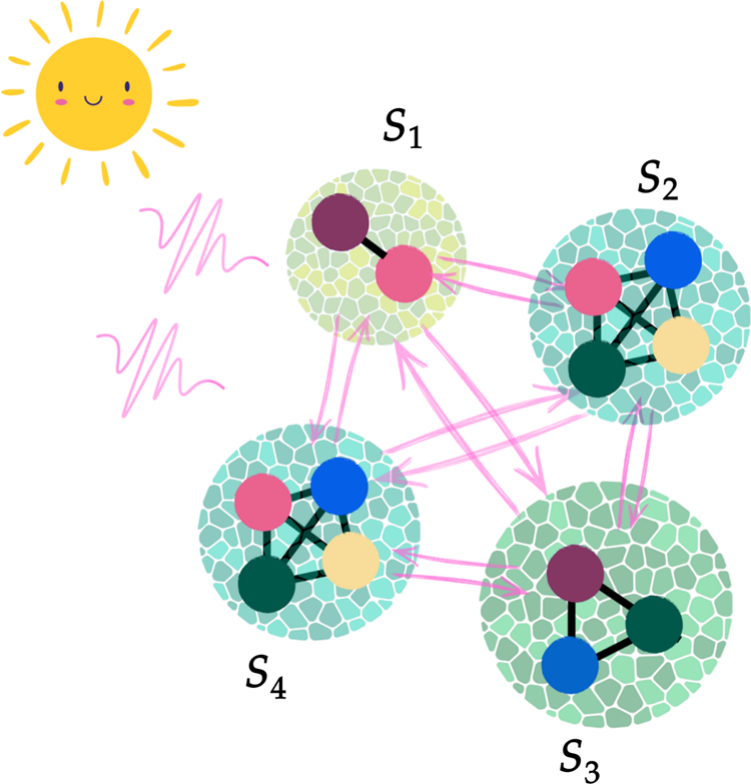
Example of the segmentation of large system into four individual segments labeled from *S*_1_ to *S*_4_. The chromophores are illustrated as small circles and solid black lines connect strongly coupled chromophores within the same segment, while weak couplings leading to energy transfer between segments are illustrated with pink arrows. Different segments are initially excited by photons (pink waves) from a light source illustrated with the sun.

The essence of the (TD-)MCFRET method is to divide a large system into smaller segments, where the energy transfer within the segments may be coherent, while the transfer between segments must be incoherent (see Figure 1). In the following, we will largely follow the description of the TD-MCFRET method from ref (18) with some generalization of the notation. The detailed steps of the derivation are given in ref (18). The Hamiltonian of a segment *S*_*i*_ of our system is given by
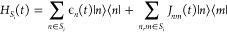
1where *n* and *m* represent different molecules in segment *S*_*i*_, ϵ_*n*_(*t*) is the time-dependent transition energy of molecule *n*, and *J*_*nm*_(*t*) is the resonance coupling between the two molecules *n* and *m*. The parameters ϵ_*n*_(*t*) and *J*_*nm*_(*t*) fluctuate in time quasi-stochastically due to interactions between the chromophores and their dynamic (thermal) environments. The time-trajectories for these quantities can be generated in different ways, for instance, by using molecular dynamics simulations combined with mappings^57^ or first-principles calculations,^58^ or by using a stochastic model, such as the Brownian oscillator model.^53^ Furthermore
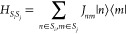
2describes the resonant interactions between the molecules in different segments. *S*_*i*_ and *S*_*j*_ (*i* ≠ *j*) denote the two interacting segments, *J*_*nm*_ is the resonance coupling between two specific chromophores. We will assume this coupling to be time-independent.^18^

In the NISE method, the time-evolution operator for the quantum states of segments *S*_*j*_ during the time step [*t*, *t* + Δ*t*] is given by
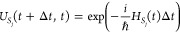
3The time-evolution for longer times is obtained by multiplying consecutive time-evolution matrices, leading to the expression
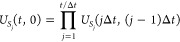
4

**Figure 2:**
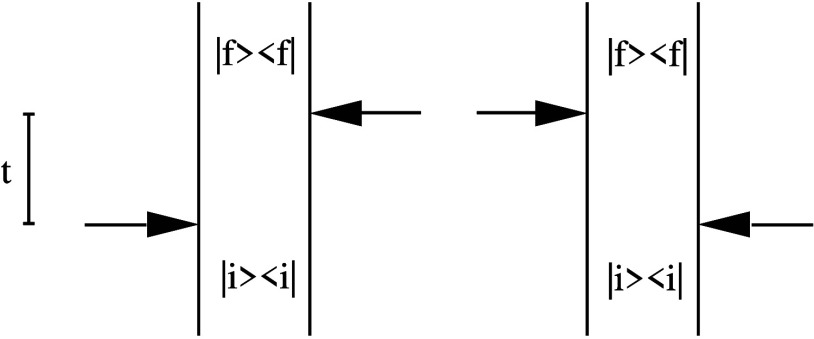
Double-sided Feynman diagrams for the time-dependent perturbation theory governing the MCFRET method. |*i*⟩ symbolize the initial state on segment *S*_*i*_, ρ_eq_^*S*_*i*_^ = |*i*⟩⟨*i*|, while |*f*⟩ is the final state on segment *S*_*j*_. The arrows symbolize the couplings between the two segments, which drive the transfer between them.

In the MCFRET method, the transfer rate between two segments is obtained using the time-dependent perturbation theory in *H*_*S*_*i*_*S*_*j*__ illustrated by the double-sided Feynman diagram in Figure 2. Each Feynman diagram can be connected with the rate response function from segment *S*_*i*_ to segment *S*_*j*_, which is defined as^18^
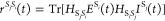
5where the trace is over the space of all (single-)exciton states in segment *S*_*j*_. Here, *I*^S_*j*_^(*t*) and *E*^S_*i*_^(*t*) are absorption and emission matrices, respectively, with their elements defined by
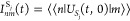
6and
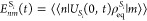
7where the outer angular brackets ⟨···⟩ denote the ensemble average over the (thermal) bath fluctuations. The thermal equilibrium density matrix, ρ_eq_^*S*_*i*_^, for segment *S*_*i*_ is
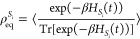
8with , where *k*_B_ is the Boltzmann’s constant and *T* is the temperature. This, thus, implies the assumption of thermalization within the segments before the transfer takes place. We will discuss the high-temperature limit as the situation where the density matrix has an equal population for all sites within a given segment. The transfer rate from segment *S*_*i*_ to *S*_*j*_ is given by the integral of the rate response function from time zero to infinity. In practice, the response function will lose coherence and decay to zero within a short time, typically in the order of one ps. This allows us to replace the upper integration boundary by a fixed maximal coherence time *t*_c_, which yields the energy transfer rate from segment *S*_*i*_ to *S*_*j*_ with the expression^43,44,59^
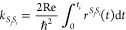
9

Since the MCFRET method only deals with incoherent processes, we introduce the decoherence rate to determine quantitatively whether this transmission process is incoherent or not. In the MCFRET implementation, the decoherence rate between segment *S*_*i*_ and *S*_*j*_ is calculated from the absolute value of the rate response function as
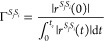
10This heuristic definition can be justified by considering an exponentially decaying rate response function. The integral in the denominator will equal the time scale of the rate response decay times a prefactor that depends on the couplings. The numerator cancels this prefactor, leaving the rate as the inverse of the time scale of the exponential decay. This is a measure of how long the coherence in the rate response function persists. When the decoherence rate Γ^*S*_*j*_*S*_*i*_^ is higher than the transfer rate *k*^*S*_*j*_*S*_*i*_^ between the segments, it can be justified to assume the transfer to be incoherent.^18^ We do note that this is only a test of whether the transfer is incoherent; it does not test the validity of the other approximations of the TD-MCFRET method, as will be discussed later. A more rigorous criterion may be possible to derive from proceeding to higher-order perturbation theory.^60,61^

The TD-MCFRET method itself as derived in ref (18) does not ensure detailed balance. While accounting for the quantum nature of the environment can fix this,^44,62,63^ the NISE approach relies on a classical bath approximation. Therefore, we implemented the standard thermal correction,^64,65^ where the thermally corrected rate is given by
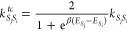
11This thermal correction was defined to ensure detailed balance while keeping the rate of equilibration between the two segments involved constant. Here, the expectation value for the energy of segment *S*_*j*_ is determined by the ensemble average (in time) over the full trajectory
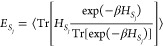
12This thermal correction can be applied independently of the high-temperature approximation for the equilibrium density matrix. When the thermal effects are already (partially) accounted for in the lineshapes, TD-MCFRET already accounts for part of the thermalization effects. To avoid overcorrection, the segment energies used in the thermal correction can be adjusted by Δ*E*_*S*_*j*__ = *k*_B_*T* ln *NP*_*S*_*j*__/*D*_*S*_*j*__, which ensures the segment equilibrium populations fulfill the detailed balance. Here, *P*_*S*_*j*__ is the equilibrium population of segment *S*_*j*_ predicted by the rates of eq 9 and *D*_*S*_*j*__ is the number of sites in segment *S*_*j*_, ensuring that Δ*E*_*S*_*j*__ = 0 when the rates are obtained in the high-temperature limit, where *P*_*S*_*j*__ = *D*_*S*_*j*__/*N*. We chose this rather simple thermalization scheme as it allows imposing the Boltzmann distribution on the segments. This and other similar schemes were previously compared in many different parameter regimes.^66^ It may be interesting to explore other alternatives^11,12,67,68^ in the future, which will require comparison with formally exact methods.

The determined rate matrices, ***k***, can be used to predict the population on different segments as a function of time given an initial population vector, *P⃗*(0).
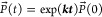
13the matrix elements of ***k*** can be given by the rates determined with or without thermal correction. The diagonal elements are given by minus the sum of the transfer rates away from the given segment, which ensures the preservation of the total population
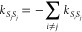
14

Assigning segments may be a bit of an art that can require different combinations of segmentation schemes. Segments may be defined explicitly by the user of the program. However, an automatic scheme was added based on cluster analysis of an absolute value density matrix (ADM) defined by the elements
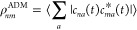
15Here, *c_na_*(*t*) is the full system wave function coefficient on site *n* for eigenfunction *a*. The average is taken over all disorder realizations along a trajectory. The absolute value kills the interference between the off-diagonal elements (coherences), which in a normal density matrix would make all coherences average to zero. If there are no eigenstates that spans both sites *n* and *m*, the ADM coherence is zero, and it is reasonable to consider the sites as belonging to different segments. In the ADM, coherences for pairs of sites contributing to the same eigenstates will be nonzero and even comparable in magnitude to the diagonal elements (populations), when the eigenstates have comparable wave function coefficients on the sites. We use cluster analysis to then assign all pairs of sites where  to the same segment. Here, ϵ is a truncation parameter. It can be adjusted to impose a looser or stricter requirement of delocalization for defining the segments. To perform cluster analysis and define distances between clusters, one may define an equivalent criterion defining the distance between two sites to be , where the truncation parameter is now ϵ_*p*_ = −ln(ϵ). A similar clustering scheme was previously defined^69^ based on the participation ratio matrix (*PR*_*nm*_ = ∑_*a*_|*c*_*na*_|^2^|*c*_*ma*_|^2^). Automatic segmentation schemes such as the one provided here will always have a heuristic element and depend on the choice of the truncation parameter. While such automated schemes are useful, it is, however, important for the user to validate the physical soundness of the resulting segmentation.

**Figure 3:**
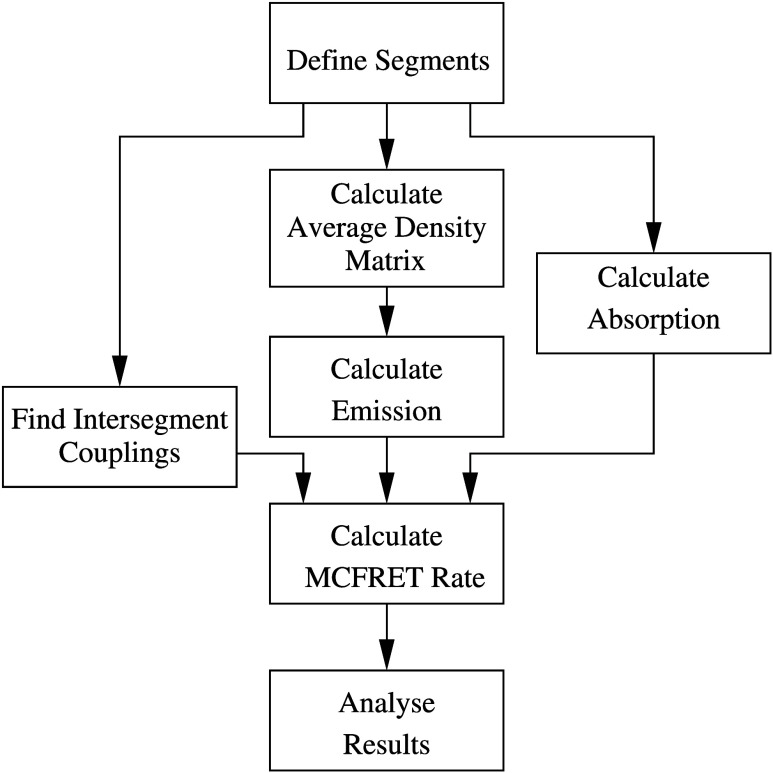
Outline of the workflow of the program. Each box represents an action that can be performed independently when the actions providing input for the box are completed. In the standard workflow, the tasks are performed in the order from top to bottom.

The overall workflow of the TD-MCFRET implementation is illustrated in Figure 3. Some tasks can be performed independently. For example, if the calculations are very time-consuming, the absorption matrix, the emission matrix, and the intersegment couplings can be determined independently of each other before the final rate calculation. For standard calculations, the program can automatically do all the needed parts sequentially. For special applications, where, for example, only the intersegment couplings differ between different calculations, those can be updated independently and only the final rate calculation needs to be repeated. This is, for example, useful for studying the distance dependence of transfer between molecular aggregates, as in such cases, only the intersegment couplings change with aggregate distance.

The TD-MCFRET method is expected to predict the transfer rate accurately when the following assumptions are met:^18^ (i) we assume the bath fluctuations on different segments to be uncorrelated, (ii) the method for calculating the line shape functions is sufficiently accurate, (iii) the population relaxation within the segments is faster than the transfer between segments, (iv) the coupling between segments is constant, and (v) the decoherence between segments is faster than the transfer between them, resulting in incoherent transfer.

## 3 Results and Discussion

In this section, we demonstrate the application of the implemented computer program by calculating the EET rate in a number of simple model systems and common systems of interest. With each system, we focus on one specific question and the analysis needed to answer it. The initial condition for the results part presented here is temperature dependent, as described by eq 8.

### 3.1 Parallel Linear Aggregates

**Figure 4:**
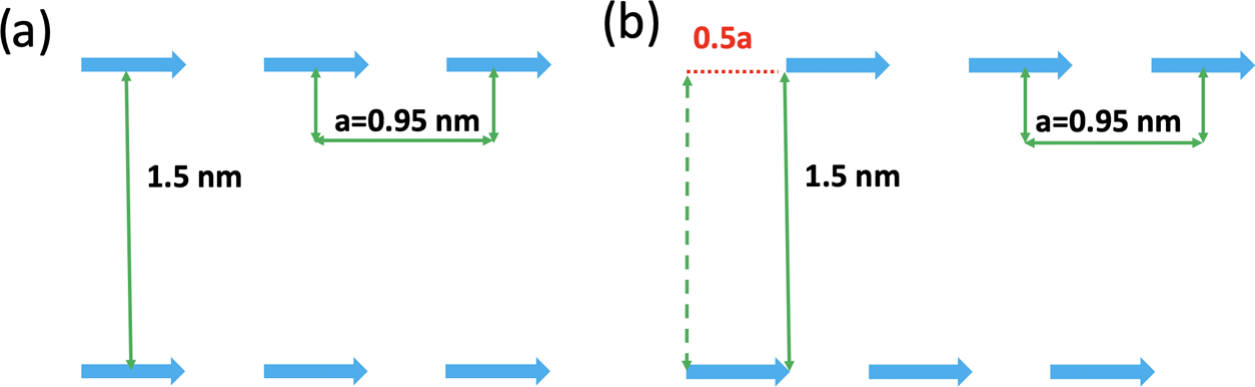
Illustration of two parallel linear J-aggregates. (a, b) show parallel aggregates with the slip between the two aggregates of 0a and 0.5a, respectively.

The exciton transfer between parallel linear aggregates was previously studied in a systematic way, comparing classical Förster theory with MCFRET.^70^ It was also found that the transfer rate depends as *R*^–2^ on the distance *R* between the aggregates at short distances and as *R*^–6^ at long distances. At short distances, exciton coherence thus leads to supertransfer.^71^ Here, we will instead examine the effect of slip on the transfer between two parallel J-aggregates as illustrated in Figure 4. The distance between the molecules inside each chain was set to a = 0.95 nm, while the distance between the linear chains was set to 1.5 nm. The slip (defined as the lateral displacement of the two nearest molecules in both chains) between the two aggregates, varied between 0a and 0.5a (0 to 0.475 nm).

Pairs of parallel J-aggregates were created. The dye molecules were given a 11.4 D transition dipole typical of cyanine dyes,^72^ all aligned along the aggregate axes. Couplings between all molecules were determined through the point-dipole coupling model. The resulting nearest neighbor coupling within one chain is −1526.1 cm^–1^, and the maximum interaction coupling between two chains without slip is 193.8 cm^–1^, while it is 122.0 cm^–1^ for 0.5a slip. We performed the simulations with different amounts of static disorder to illustrate the impact of the static disorder on the transfer rate. In all the model systems, all site energies were given a dynamic Gaussian dynamic disorder component with a standard deviation of (σ_dyn_) 1500 cm^–1^ and a correlation time of (τ_dyn_) 6 fs. This dynamic disorder is consistent with a dephasing rate of, Γ = 2πσ_dyn_^2^τ_dyn_ = 2531 cm^–1^, comparable in magnitude to the intra-aggregate nearest-neighbor coupling, but much larger than the interaggregate coupling ensure that the transfer process remains incoherent. An additional slow (static) component was added with three different choices of standard deviation (σ_static_): 750, 1500, and 3000 cm^–1^, and a much slower correlation time of (τ_static_) 10 ps. We denote these three models with different static disorder the small- medium- and large-disorder models. The disorder correlation time scales are consistent with typical values observed in cyanine aggregates.^72,73^ Disorder trajectories of 600 ps were generated with 200 molecules in each J-aggregate. The time step in the simulations was set to 3 fs and the maximal coherence time was set to *t*_c_ = 72 fs. We averaged over 10,000 equidistantly spaced disorder realizations along the generated trajectories for calculating the absorption and emission matrices along the trajectory. A temperature of 300 K was used during the simulation process.

**Figure 5:**
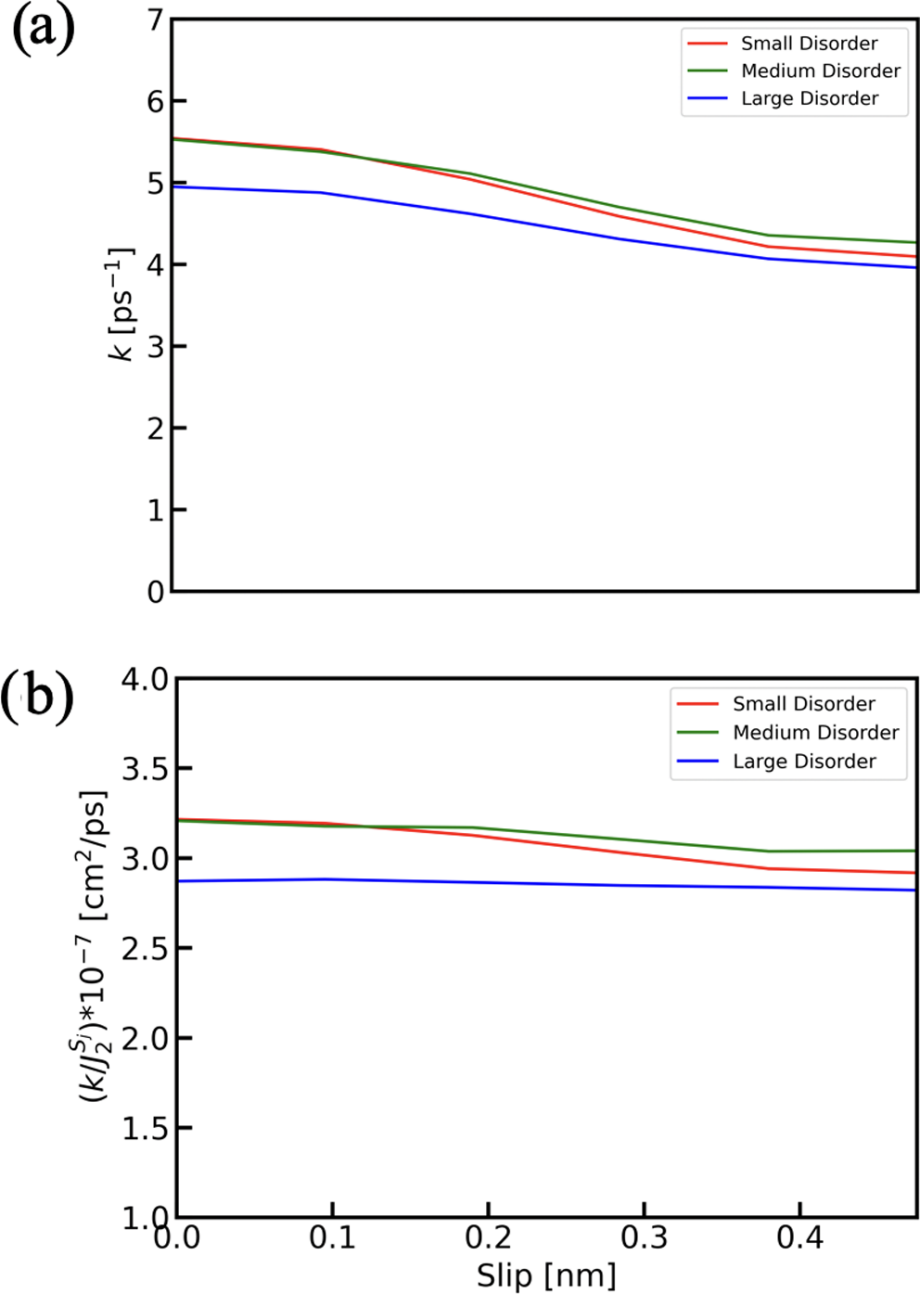
(a) Slip-dependent rate between two J-aggregates separated by 1.5 nm with different degrees of disorder. (b) The transfer rate divided by the sum of the squared couplings between a site in one aggregate and the sites in the other aggregate varies with slip.

Figure 5a shows the effects of the slip on the transfer rate for several magnitudes of the static disorder. For each disorder case, the EET rate is seen to decrease with the growing value of the slip (until the maximum slip of 0.5a). This simply results from the fact that a larger slip leads to larger intermolecular distances between the closest molecules in the two aggregates, which in turn leads to smaller interactions. A slip of 0.5a results in a reduction of the transfer rate by about 25, 29, and 35% compared to no slip for the large, medium, and small disorder cases, respectively.

The results also reveal the effects of static disorder on the energy transfer between the two chains. In general, the increasing static disorder causes the system to become more localized, leading excitons to become confined to individual sites or small clusters of neighboring molecules. In this large-disorder case, the transfer is expected to be in the conventional Förster limit, with the transfer occurring predominantly between independent chromophores. In that case (i.e., ignoring exciton delocalization within the aggregates), the transfer rate should be described by the sum of the conventional Förster energy transfer rates and is thus proportional to the average of the sum of the squares of the couplings of one site in one aggregate with all the chromophores on the other J-aggregate^70^
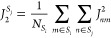
16To characterize the effect of delocalization, we evaluated the ratio between the transfer rate and this quantity. In Figure 5b, one sees the variation in the ratio (*k*/*J*_2_^*S*_*j*_^) with the increase in slip. As anticipated, the large-disorder case, where the excitons are more localized, tends to follow the relationship of the rate being proportional to the sum of the squares of the couplings more closely compared to the other disorder cases resulting in a horizontal line in Figure 5b. For smaller static disorder, the effect of supertransfer^71^ is seen, and the transfer rates do not follow the conventional Förster behavior. At large aggregate separations, one would expect the transfer rate to be independent of the slip in all cases.

In general, one will expect that when the static disorder is increased, the EET rate will decrease. However, when the disorder amplitude is comparable to the coupling strength in a large molecular system, the dynamics become more nuanced. At zero slip (Figure 5a), the transfer rates in the small- and medium-disorder cases are quite similar, with the small disorder case showing only a slightly faster rate than the medium disorder. As the slip increases, the transfer rate in the medium disorder case surprisingly becomes faster than in the small disorder case. This interesting effect must arise from the complex interplay between changing the delocalization along the chains and increasing the disorder between chains. We find that for slips of 0.2a and larger all couplings between the two aggregates except the largest one are negative. In particular for zero slip, the second largest coupling is 16.5 cm^–1^ while it is −31.3 cm^–1^ for the slip of 0.5a. This suggests that there may be an effect of destructive interference in the supertransfer,^71^ which is larger for the slipped configurations. Such an effect is expected to be enhanced by increasing delocalization. The delocalization length using the inverse participation ratio measure^74^ for the given parameters was found to be 3.8, 7.0, and 10.2 molecules within each individual aggregate for large, medium, and small disorders, respectively. For the small disorder, the effect of destructive interference between transfer pathways can thus be expected to be larger. This may be an interesting topic for further study.

In practice, a wider variety of stacking arrangements can be found in, for example, two-dimensional films.^75^ This is beyond the scope of the present paper, but the above example illustrates the variations of slip on the transfer rate between two parallel J-aggregates with the TD-MCFRET method and how it can be analyzed with the new program.

### 3.2 Transfer between Rings

**Figure 6:**
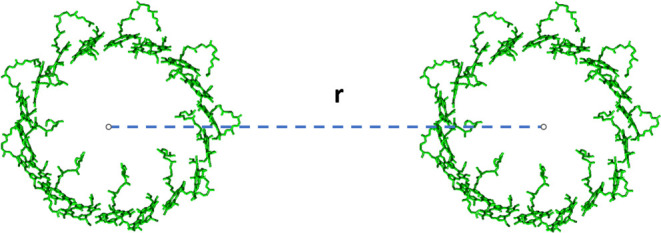
Schematic overview of B850 chromophores in two LH2 complexes of *R. acidophilus*. In each complex, the B850 bacteriochlorophylls form a ring of diameter ∼60 Å. In our calculation, the center-to-center distance of the two LH2 complexes, *r*, was varied from 75 to 600 Å. The picture was rendered using VMD.^80^

The energy transfer between the LH2 rings of purple bacteria^76^ has previously been studied with different methods,^27,28,77^ including other MCFRET implementations.^18^ Here, we will apply the Hamiltonian used in our previous paper.^18^ We increase the ring-to-ring distance, *r*, from 75 to 600 Å and examine the short-range breakdown of the conventional Förster transfer regime, which would predict a simple 1/*r*^6^ scaling of the energy transfer rate. The typical center-to-center distance between neighboring LH2 complexs in a natural system is 75 to 80 Å.^78,79^ Each LH2 system consists of two rings of bacteriochlorophyll (BChl) molecules, which are named the B850 ring and B800 ring based on the absorption wavelength of the two resulting bands.^53,81^ The high-frequency ring contains 8 to 12 BChl molecules depending on the bacterial species, leading to the B800 band. The low-frequency ring contains twice as many BChl molecules as the high-frequency ring, and its absorption is red-shifted to 850 nm, resulting in the B850 absorption band. Here, we focus on the *Rhodoblastus acidophilus* (previously known as *Rhodopseudomonas acidophila*(76)) bacteria and the B850 rings depicted in Figure 6, which only contain 18 B850 BChl molecules.

We focused on the transfer between the B850 rings, and we employed the simplest possible Frenkel exciton Hamiltonian for these chromophores while neglecting the B800 chromophores. The general form of the Hamiltonian is shown in eqs 1 and 2. The parameters were identical to those used in ref (18). We began with the crystal structure taken from the 1kzu protein data bank file.^82^ The B850 chromophores were given an average transition frequency of 11,955 cm^–1^.^18^ All chromophores were coupled to an overdamped Brownian oscillator bath with a 150 fs correlation time and disorder magnitude σ = 256 cm^–1^. The time step was set to 3 fs and the length of the trajectories was 200 ps. The resonant couplings were determined using the TrEsp model with the transition charges calculated by the TDDFT/B3LYP method.^83−85^ We included a scaling factor in the couplings resulting from the dielectric screening of 1/ε_r_ = 0.55 as previously used.^18^ The expectation value for the energy of the two rings was identical; therefore, the thermal correction was neglected in this case (eq 11). The temperature for the thermal equilibrium of the emission was set to 300 K.

**Figure 7:**
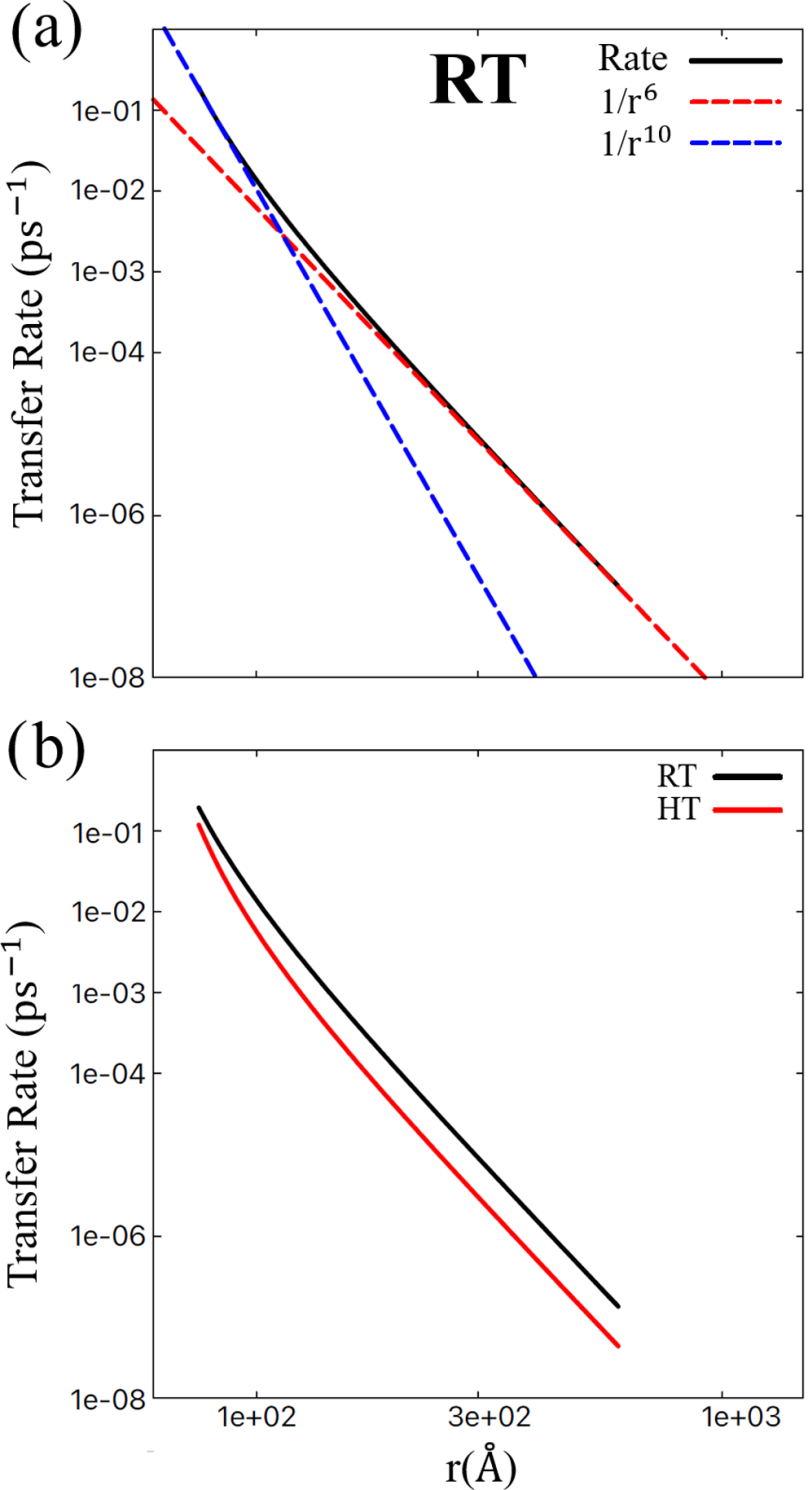
Double-logarithmic plot of the transfer rate as a function of the distance between two B850 rings. (a) RT (300 K) data along with straight lines corresponding to 1/*r*^6^ and 1/*r*^10^ scaling with distance in the long- and short-distance regimes, respectively. (b) A comparison of the EET transfer rate as a function of distance at RT and HT limit.

In Figure 7a, one can see that the EET rate scales according to 1/*r*^10^ at the shortest distances, while it is proportional to 1/*r*^6^ at distances beyond 200 Å, as expected in the Förster limit.^41,42^ The reason for this difference can be understood in the following way. The distance between two neighboring B850 pigments is ∼9 Å,^18,86^ resulting in strong delocalization of the exciton states in the B850 ring. For long distances, one may apply the point-dipole approximation to the entire aggregate, meaning that only superradiant states matter. This indeed results in a 1/*r*^6^ behavior for the incoherent energy transfer rate. However, at short distances, comparable to the diameter of the ring, the optically dark excitons also affect the transfer process. Higher-order multiples will then start to play a role. The lowest-order correction will be of dipole–quadrupole nature, which would give a 1/*r*^8^ behavior for the rate.^87,88^ The next order is due to quadrupole–quadrupole interactions, which would agree with the observed 1/*r*^10^ short-distance dependence of the rate.^89,90^ The fact that the fit only shows that the latter may be a consequence of the limited fit range, but may also be a real effect. For ordered ring aggregates dipole–quadrupole interaction connects states in the two rings that are not resonant with each other, which will reduce the transfer rate, while quadrupole–quadrupole interactions are resonant.

In Figure 7(b), we compare the room temperature (RT) results with the high-temperature (HT) limit results. In the HT limit, the EET rate is slower. We simply compare where the rates for the shortest distance considered, 75 Å, the EET rate at RT is 0.191 ps^–1^ while it is 0.117 ps^–1^ in the HT limit. This can be understood from the fact that at RT, the equilibrium population in each ring has a larger weight on low-energy superradiant states than at HT. Furthermore, dark states (relatively) contribute, which have a higher population in the HT limit.^91^ The observed behavior is similar to that previously reported in refs (91−93), where rates in the order of 0.1–0.4 ps^–1^ were found for closely packed LH2 complexes, depending on the choice of variation of parameters and temperature. These studies further provided insight into the optimal disorder and symmetry for the transfer.

### 3.3 Coherent vs Incoherent Transfer in FMO

The energy transfer processes in the Fenna-Matthews-Olson (FMO) complex were studied in detail experimentally.^5^ For this system, the long-standing debate on the role of coherent vs incoherent transfer has essentially settled the transfer as being predominantly incoherent, at least at physiologically relevant temperatures.^1^ Here, we demonstrate how this transfer can be examined theoretically using the MCFRET method. To achieve this, we used the seven-site exciton Hamiltonian from ref (5) and the Brownian oscillator model for the disorder from ref (94), which demonstrated good agreement with 2DES experiments.^95^

We first considered the situation where all sites are treated as independent segments. The decoherence rate (according to eq 10) between individual sites is ∼14.3 ps^–1^ for all pairs. This is a result of the sites all having the same disorder. The resulting transfer rate between the different Bchl molecules is reported in Table 1. Comparing the predicted transfer rates clearly shows that the transfer between the pairs (4,7) and (1,2) must have a significant coherent contribution, as the downward transfer rate is larger than or the same as the decoherence rate. For the pair (5,6), the transfer rate is also quite close to the decoherence rate, justifying treating these two sites as a segment. The pair (4,5) also has a large transfer rate compared to the decoherence, which will be discussed later.

**Table 1:** Thermally Corrected Transfer Rates in ps^–1^ for FMO at 77 K with All Sites Defined as Individual Segments^a^

BChl	From 3	From 4	From 7	From 1	From 5	From 6	From 2
To 3	–0.240	6.650	0.098	0.015	0.000	0.005	0.023
To 4	0.239	–11.053	**17.147**	0.111	**14.216**	0.502	0.016
To 7	0.001	3.392	–18.061	0.092	0.024	4.823	0.002
To 1	0.000	0.015	0.063	–2.987	0.215	0.347	**34.592**
To 5	0.000	0.980	0.008	0.109	–18.741	**9.672**	0.019
To 6	0.000	0.015	0.745	0.078	4.284	–15.689	1.032
To 2	0.000	0.000	0.000	2.581	0.003	0.341	–35.683

a The sites are sorted according to their average exciton energy.

**Figure 8:**
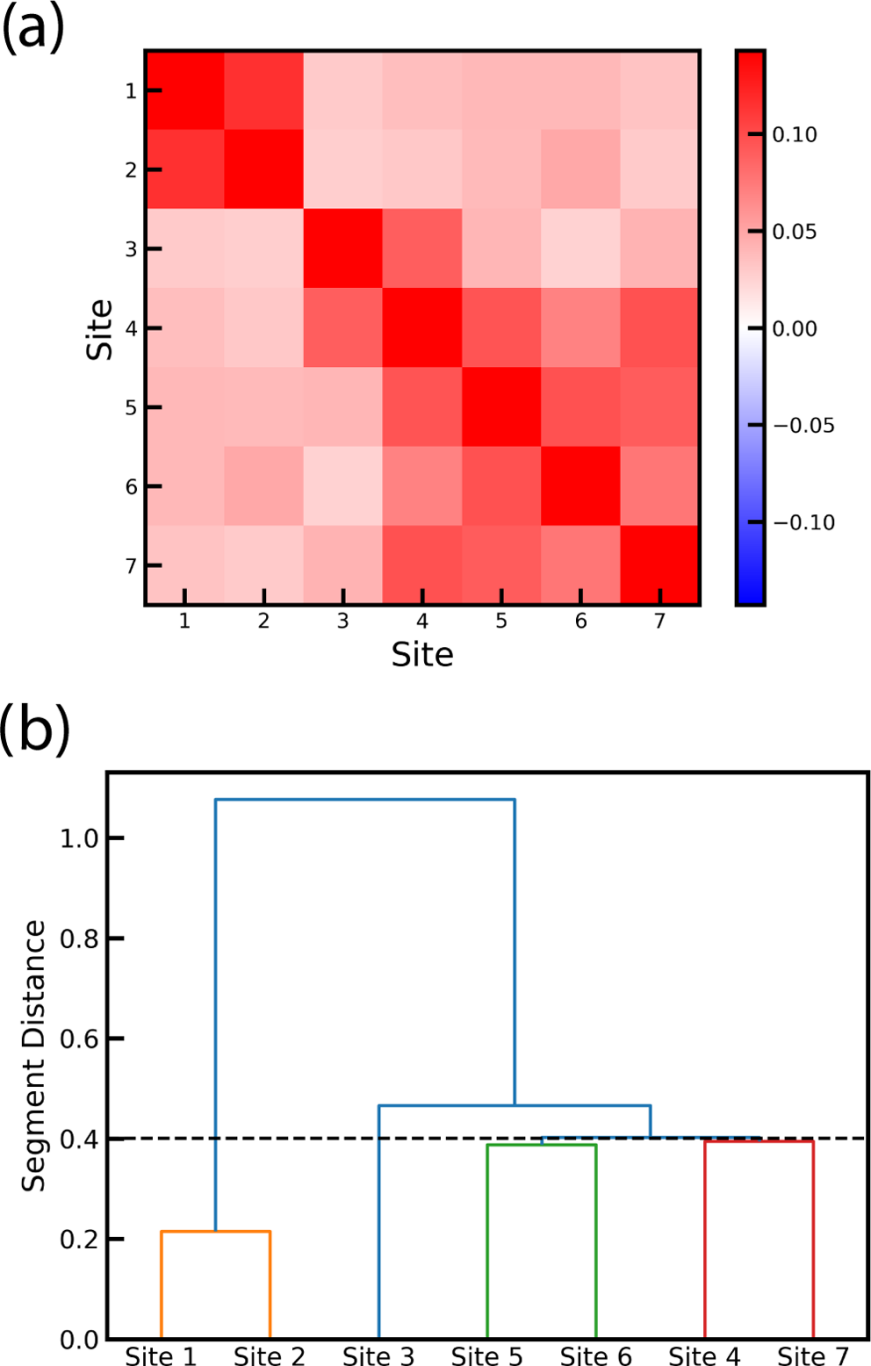
(a) The ADM for the FMO model normalized to the diagonal values. (b) A dendrogram based on the ADM using the criterion of segment distances. The cutoff parameter, indicated by the dashed vertical line, ϵ_*p*_ = 0.4, was used to define the clusters considered to obtain the results in Table 2.

The ADM (eq 15) for the FMO model is presented in Figure 8 together with a dendrogram analysis. This illustrates which sites are most strongly connected. We then defined four segments numbered from one to four, sorted by the expectation value for the energy for the segment. For this, we used a cutoff of ϵ_*p*_ = 0.4, resulting in BChl molecules assigned as strongly coupled in agreement with the assignment in ref (5), which is assigned as a canonical segmentation scheme. Segment 1 contains BChl 3, segment 2 BChl 4 and 7, segment 3 BChl 1 and 2, while segment 4 contains BChl 5 and 6. This assignment is also in line with our findings above; even from that, one could also consider treating sites 4 through 7 as one segment. One could choose a larger cutoff, which would merge segments 2 and 4.

**Table 2:** Thermally Corrected Transfer Rates in ps^–1^ for FMO at 77 K^a^

segment	From 1	From 2	From 3	From 4
To 1	–0.493	6.627	0.223	0.001
To 2	0.491	–7.246	0.035	11.890
To 3	0.002	0.006	–0.330	0.296
To 4	0.000	0.612	0.072	–12.187

a The segments were identified using the dendrogram of Figure 8b and are sorted according to their average exciton energy.

Table 2 shows the transfer between the defined segments. For the transfer from segment 4 to segment 2, the decoherence is, however, only slightly faster than the transfer, consistent with the above comment that a slightly larger cutoff distance in the ADM would merge these two segments. At physiologically relevant temperatures, the frequency fluctuations are much larger, and therefore, the decoherence rates are faster.

The observed decoherence rates in Table 3 are not symmetric. The decoherence calculated between emission of, for example, site 3 with absorption of site 2 is not identical to decoherence between emission from site 2 and absorption from 3. This asymmetry arises from the assumption of thermalization within the segments before the transfer takes place, as accounted for by the density matrix in eq 8.

**Table 3:** Decoherence Rates in ps^–1^ for FMO at 77 K

segment	From 1	From 2	From 3	From 4
To 1	0.0	17.2	16.7	14.2
To 2	18.4	0.0	21.4	16.2
To 3	20.1	13.5	0.0	20.2
To 4	15.3	16.4	20.5	0.0

**Figure 9:**
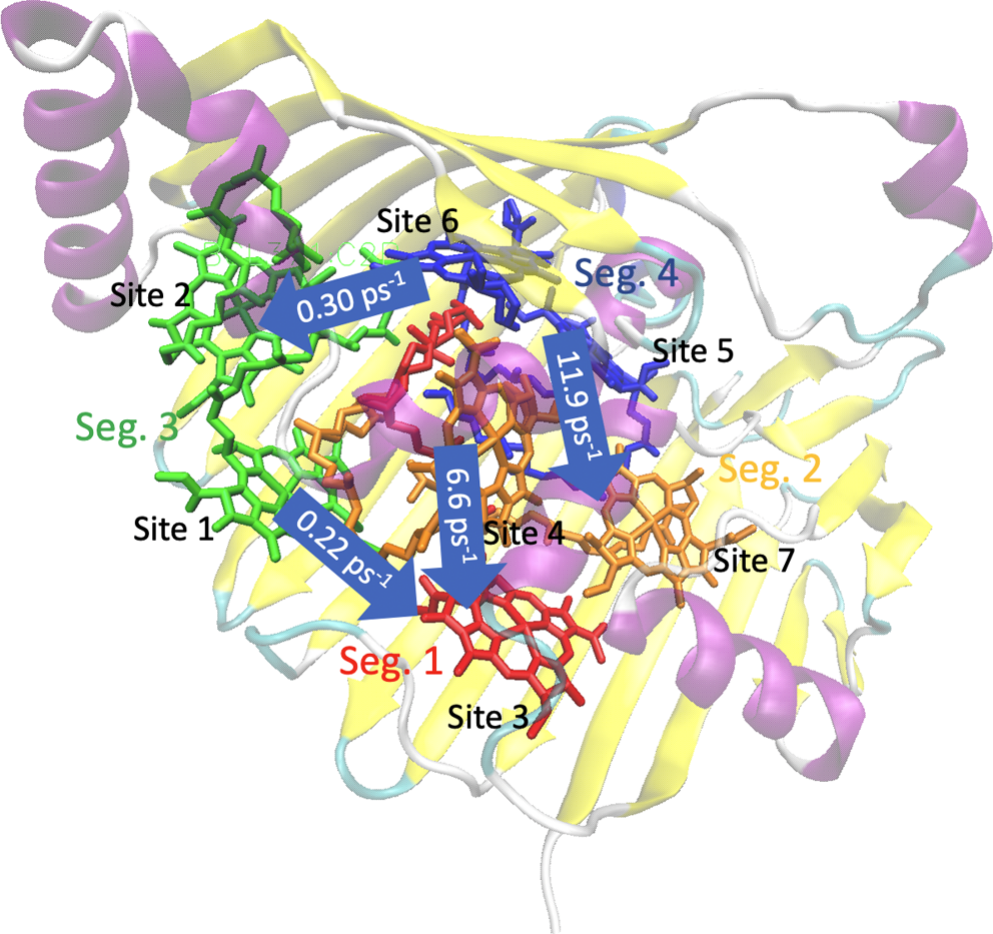
Predicted energy transfer between the four canonical segments of FMO. The protein scaffold is shown with β-sheets as yellow arrows and α-helices as purple coils. The BChl molecules are illustrated in colors indicating their segment. Different sites are labeled from site 1 to site 7. The arrows show the largest rates connecting the different segments.

The transfer pathway revealed from the calculations is in accordance with the experimental observations,^5^ where the excitation reaches segments 3 and 4 from the baseplate,^76^ and is transferred through segment 2 or directly to segment 1 following a downhill energy transfer pathway^1^ as illustrated in Figure 9.

**Figure 10:**
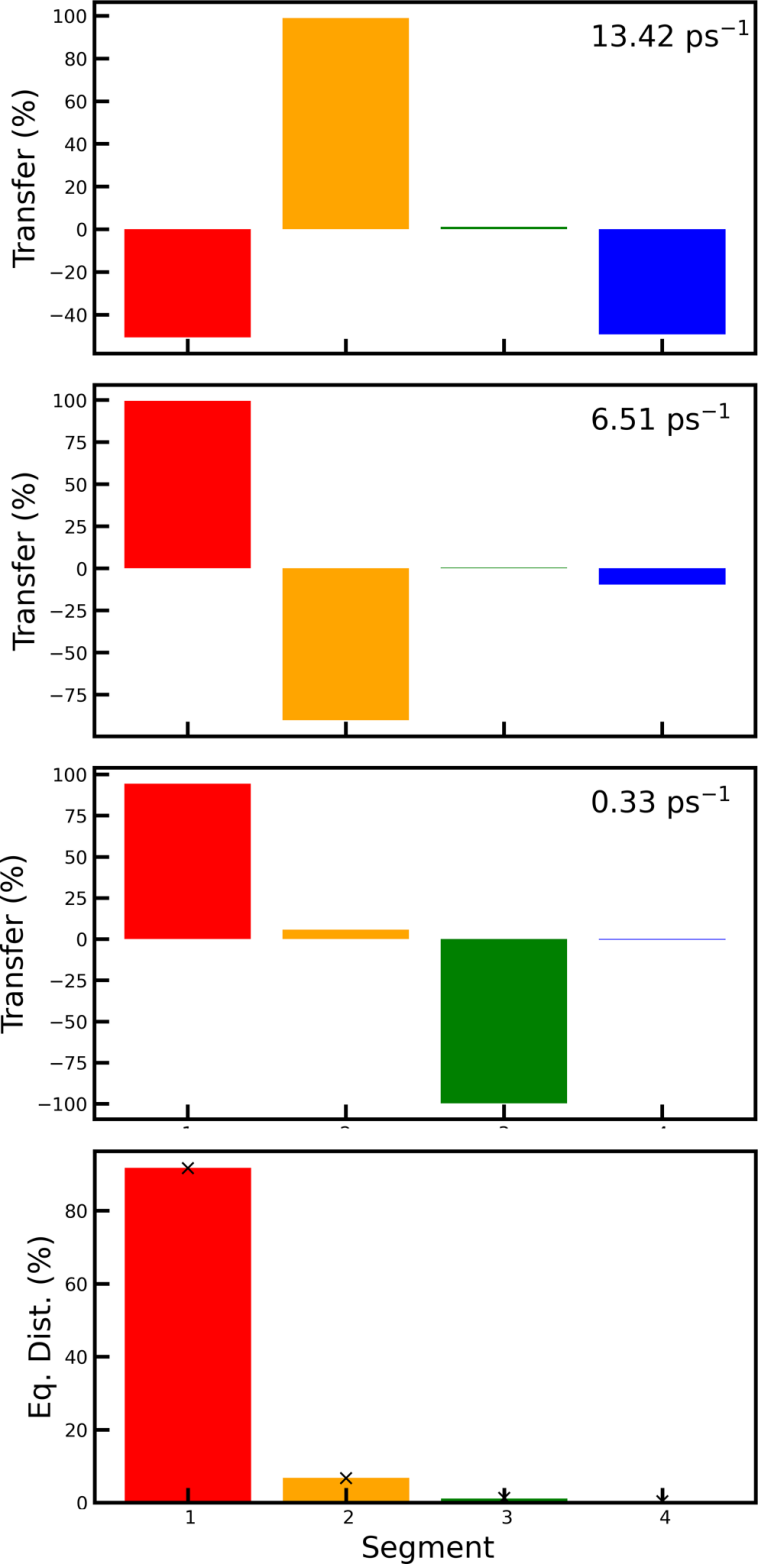
First three panels show the three relaxation processes through the normalized eigenvectors connected with each of the three nonzero eigenvalues of the rate matrix. The bottom panel shows the predicted equilibrium populations for the four segments identified in the FMO complex after the thermal correction, which was calculated at 77 K. The crosses in the bottom panel indicate the predictions using the Boltzmann distribution.

The exciton transfer paths in FMO are illustrated in Figure 9. For further analysis, the thermally corrected transfer rate matrix was diagonalized. This gives four eigenvectors, where the one connected with an eigenvalue near zero contains the information on the equilibrium populations on the segments. In Figure 10, these populations are shown, and it is clear that the vast majority will end up in segment 1 at equilibrium, which has the lowest energy and is closest to the reaction center. The populations predicted by the Boltzmann distribution are also illustrated. As expected, these are seen to match with the values coming from the thermally corrected rate matrix, as this was imposed with the thermal correction procedure of eq 11.

The three remaining eigenvectors reveal the possible relaxation pathways back to equilibrium and the eigenvalues are the corresponding relaxation rates, which are the rates that can be experimentally observed. These processes are shown in Figure 10. The fastest process brings the population from segment 1 and 4 to segment 2 (or vice versa). This process has a 13.4 ps^–1^ rate constant. The next process predominantly brings the population from segment 2 to segment 1 (or vice versa) and does so with a rate of 6.51 ps^–1^. The slowest process takes the population from segment 3 to segment 1 (or vice versa). This happens at a much slower rate of 0.33 ps^–1^.

Energy transfer in FMO has been studied very extensively,^1^ both theoretically and experimentally. Recent theory demonstrated picosecond scale decay of energy transfer similar to ours even though no explicit rate matrices were provided, preventing a more direct comparison.^93,96^ A recent publication^1^ similarly suggests the presence of both fast ∼10 ps^–1^ rates as well as slower relaxation through segment 3 to the lowest energy segment 1 while using a more detailed spectral density for the bath than employed here. Compared with recent experiments,^97^ our theory also gives reasonable rates. Both referenced theoretical predictions^1^ and experimental^97^ results indicate that sites 1 and 2 (referred to here as segment 3) are the weakest connected with the rest of the system. Our rate of decay out of this segment is 0.33 ps^–1^, while 0.83 ps^–1^ is reported from the experiment. The fastest rate in the experiment is 16 ps^–1^, which is only slightly faster than we find for the reequilibration between segment 2 and segments 1 and 3. The reported fits to the recent experimental data^97^ do give significant deviations from previous fits to experimental data^98^ and variation between experiments can be expected depending on fitting procedures, bacterial species, growth conditions, and isolation techniques. For a direct comparison it would be more instructive to calculate actual time dependent spectra and compare the spectral evolution directly.^99^

In summary, the FMO simulations show that energy transfer between sites 1 and 2, between sites 5 and 6, and between sites 4 and 7 must be treated as being coherent at 77 K. The division of sites 4, 5, 6, and 7 into two separate segments (here segments 2 and 4) is on the edge and the transfer between these segments is only about 3 times slower than the dephasing between them. The cutoff in the dendrogram (Figure 8b) also shows that merging sites 4 through 7 in one segment would be reasonable. Overall, the analysis does show the expected transfer from the segments with the highest energy, which are closest to the baseplate, down to the segment with the lowest energy, which is also closer to the reaction center, where the energy eventually has to go. This is in line with previous findings.^1,5^ At physiological temperatures, the energy disorder is about twice as large and the transfer is significantly more incoherent.^38^

## 4 Implementation Details

The TD-MCFRET implementation uses openMP parallelization for the propagation needed in eqs 6 and 7, where consecutive matrix multiplications are needed. For large systems, this means that the propagation for each site can be treated on a separate CPU. The number of sites should thus preferably be divisible by the number of openMP threads. The matrix products in eq 5 are running over the number of sites and these matrix multiplications are also made parallel with openMP. The LAPACK library^100^ is used for the matrix diagonalization needed to determine the density matrices. This matrix diagonalization can, therefore, also be done in parallel using the LAPACK openMP settings.

The numerical integration of the rate response function was analyzed by both using the trapezium sum and the Simpson 1/3 rule for the function *R*(*t*) = exp(−*t*^2^)cos(*t*), which resembles a typical rate response function shape, but where the exact integral is known. It was found that the trapezium sum is converging faster than the Simpson 1/3 rule for this integral. Furthermore, the cutoff of the integral at a finite time instead of at infinite time can be partially compensated by using a weight of one for the last data point instead of one-half as given by the trapezium sum. The implemented program both calculates the trapezium sum and the Simpson 1/3 rule and provides a warning when the difference between these is larger than a few percent. Possible integration issues can be solved by using smaller timesteps.

The rate matrix predicted by the program may have complex eigenvalues. This corresponds to the presence of an (unphysical) circular current, which can occur for three or more segments when detailed balance is not fulfilled. We found that averaging over more samples typically solves this issue. An eigenstate analysis is implemented in the program, and the user is issued a warning if complex eigenvalues are detected. If the imaginary components are small enough to be considered numerical noise, it may still be sensible to use the resulting rate matrices with caution.

In the present calculations, we treated static disorder as slow dynamic disorder. This approach simplifies the calculations, however, when the static disorder is significantly large compared to the coupling and dynamic disorder, this approach will predict a single average rate, while in reality, there will be a broad distribution of rates. Strong static disorder can, however, still be treated using the implemented code. This requires the user to generate a large number (in the order of 1000) of trajectories with explicit static disorder. A separate rate is then calculated for each trajectory. The rate distribution can then be analyzed. Furthermore, if integrating with the CG-2DES spectral calculation scheme previously implemented in NISE,^99^ spectra can be calculated for each disorder trajectory before averaging the spectra. In this limit, one also needs to be cautious with the segment definition, which one may want to adapt for each disorder trajectory. The developed automatic segmentation scheme may come in particularly handy in this case.

## 5 Conclusions

We have demonstrated the implementation of the TD-MCFRET algorithm in the NISE program for the calculation of exciton energy transfer rates. The important functionalities include the ability of the program to help define physically meaningful segments using the absolute value density matrix, the calculation of transfer rates between segments, and the ability to help judge to what extent the transfer is actually coherent. The program provides postprocessing options that allow the imposing of a thermal correction to make the equilibrium populations obey the Boltzmann distribution. Furthermore, an analysis of the transfer pathways through the eigenvalues and eigenvectors of the rate matrix is provided. The overall implementation of the TD-MCFRET method in the NISE program allows the users to calculate the EET rate and analyze the EET process systematically and efficiently.

The new program was first demonstrated on a parallel linear aggregate system, where we examined the effect of slip and disorder strength on the transfer rate. Subsequently, we investigated the excitation energy transfer rate between two B850 rings and studied its variation with distance. Further exploration of the FMO complex at low temperatures underscores the efficacy of our approach in capturing incoherent energy transfer processes within the FMO complex employing a segmentation scheme. The findings were demonstrated to be in agreement with previous calculations and experiments. The provided analysis further connected well with physical intuition. In particular, a scheme for automatically assigning segments was demonstrated, and a test was provided for the applicability of the incoherent approximation.

The presented implementation of the TD-MCFRET program provides a way of determining rates and segments needed for newly developed coarse-grained computational spectroscopic methods for two-dimensional spectroscopies.^7,99^

## Data Availability

The data that support the findings of this study are available from the corresponding author upon reasonable request. The developed computer code is available on the GitHub page^20^https://github.com/GHlacour/NISE_2017 and the python scripts for the cluster analysis as well as input files for the FMO example are available on the GitHub page^101^https://github.com/GHlacour/NISE_Tutorials.

## References

[ref1] Cao J.; Cogdell R. J.; Coker D. F.; Duan H.-G.; Hauer J.; Kleinekathöfer U.; Jansen T. L. C.; Mančal T.; Miller R. J. D.; Ogilvie J. P.; Prokhorenko V. I.; Renger T.; Tan H.-S.; Tempelaar R.; Thorwart M.; Thyrhaug E.; Westenhoff S.; Zigmantas D. Quantum Biology Revisited. Sci. Adv. 2020, 6, eaaz4888 10.1126/sciadv.aaz4888. 32284982 PMC7124948

[ref2] Scholes G. D.; Fleming G. R.; Chen L. X.; Aspuru-Guzik A.; Buchleitner A.; Coker D. F.; Engel G. S.; Van Grondelle R.; Ishizaki A.; Jonas D. M.; Lundeen J. S.; McCusker J. K.; Mukamel S.; Ogilvie J. P.; Olaya-Castro A.; Ratner M. A.; Spano F. C.; Whaley K. B.; Zhu X. Using Coherence to Enhance Function in Chemical and Biophysical Systems. Nature 2017, 543, 647–656. 10.1038/nature21425. 28358065

[ref3] Zhang C.; Yan Y.; Zhao Y. S.; Yao J. From Molecular Design and Materials Construction to Organic Nanophotonic Devices. Acc. Chem. Res. 2014, 47, 3448–3458. 10.1021/ar500192v. 25343682

[ref4] Berera R.; van Grondelle R.; Kennis J. T. Ultrafast transient absorption spectroscopy: principles and application to photosynthetic systems. Photosynth. Res. 2009, 101, 105–118. 10.1007/s11120-009-9454-y. 19578970 PMC2744833

[ref5] Brixner T.; Stenger J.; Vaswani H. M.; Cho M.; Blankenship R. E.; Fleming G. R. Two-Dimensional Spectroscopy of Electronic Couplings in Photosynthesis. Nature 2005, 434, 625–628. 10.1038/nature03429. 15800619

[ref6] Biswas S.; Kim J.; Zhang X.; Scholes G. D. Coherent two-dimensional and broadband electronic spectroscopies. Chem. Rev. 2022, 122, 4257–4321. 10.1021/acs.chemrev.1c00623. 35037757

[ref7] Jansen T. L. C. Computational Spectroscopy of Complex Systems. J. Chem. Phys. 2021, 155, 170901 10.1063/5.0064092. 34742221

[ref8] Redfield A. G. On the Theory of Relaxation Processes. IBM J. Res. Dev. 1957, 1, 19–31. 10.1147/rd.11.0019.

[ref9] Novoderezhkin V. I.; Palacios M. A.; van Amerongen H.; van Grondelle R. Energy-Transfer Dynamics in the LHCII Complex of Higher Plants: Modified Redfield Approach†. J. Phys. Chem. B 2004, 108, 10363–10375. 10.1021/jp0496001. 16852271

[ref10] Häse F.; Kreisbeck C.; Aspuru-Guzik A. Machine Learning for Quantum Dynamics: Deep Learning of Excitation Energy Transfer Properties. Chem. Sci. 2017, 8, 8419–8426. 10.1039/C7SC03542J. 29619189 PMC5863613

[ref11] Jansen T. L. C. Simple Quantum Dynamics with Thermalization. J. Phys. Chem. A 2018, 122, 172–183. 10.1021/acs.jpca.7b10380. 29199829 PMC5770886

[ref12] Holtkamp Y.; Kowalewski M.; Jasche J.; Kleinekathöfer U. Machine-Learned Correction to Ensemble-Averaged Wave Packet Dynamics. J. Chem. Phys. 2023, 159, 094107 10.1063/5.0166694. 37671967

[ref13] Beck M. The Multiconfiguration Time-Dependent Hartree (MCTDH) Method: A Highly Efficient Algorithm for Propagating Wavepackets. Phys. Rep. 2000, 324, 1–105. 10.1016/S0370-1573(99)00047-2.

[ref14] Tanimura Y. Numerically “Exact” Approach to Open Quantum Dynamics: The Hierarchical Equations of Motion (HEOM). J. Chem. Phys. 2020, 153, 020901 10.1063/5.0011599. 32668942

[ref15] Suess D.; Eisfeld A.; Strunz W. T. Hierarchy of Stochastic Pure States for Open Quantum System Dynamics. Phys. Rev. Lett. 2014, 113, 150403 10.1103/PhysRevLett.113.150403. 25375694

[ref16] Gera T.; Chen L.; Eisfeld A.; Reimers J. R.; Taffet E. J.; Raccah D. I. G. B. Simulating Optical Linear Absorption for Mesoscale Molecular Aggregates: An Adaptive Hierarchy of Pure States Approach. J. Chem. Phys. 2023, 158, 174103 10.1063/5.0141882. 37125709

[ref17] Mukai K.; Abe S.; Sumi H. Rapid Excitation-Energy Transfer to Optically Forbidden States in Light-Harvesting Antennas of Photosynthetic Bacteria. J. Lumin. 2000, 87–89, 818–820. 10.1016/S0022-2313(99)00425-1.

[ref18] Zhong K.; Nguyen H. L.; Do T. N.; Tan H.-S.; Knoester J.; Jansen T. L. C. An Efficient Time-Domain Implementation of the Multichromophoric Förster Resonant Energy Transfer Method. J. Chem. Phys. 2023, 158, 064103 10.1063/5.0136652. 36792497

[ref19] Jang S.; Silbey R. J. Single Complex Line Shapes of the B850 Band of LH2. J. Chem. Phys. 2003, 118, 9324–9336. 10.1063/1.1569240.

[ref20] See: https://github.com/GHlacour/NISE_2017 for NISE_2017 (accessed July 2, 2024).

[ref21] Zhuang W.; Abramavicius D.; Venkatramani R.; Jansen T. L. C.; Voronine D.; Robinson B.; Hayashi T.; Mukamel S. The Mukamel Group Software, SPECTRON. https://mukamel.ps.uci.edu/software.html. (Accessed February 2, 2022).

[ref22] Anda A.; Hansen T.; De Vico L. Multireference excitation energies for bacteriochlorophylls a within light harvesting system 2. J. Chem. Theory Comput. 2016, 12, 1305–1313. 10.1021/acs.jctc.5b01104. 26796483

[ref23] Ishizaki A.; Fleming G. R. Quantum coherence in photosynthetic light harvesting. Annu. Rev. Condens. Matter Phys. 2012, 3, 333–361. 10.1146/annurev-conmatphys-020911-125126. 22753820

[ref24] Zhang Z.; Lambrev P. H.; Wells K. L.; Garab G.; Tan H.-S. Direct observation of multistep energy transfer in LHCII with fifth-order 3D electronic spectroscopy. Nat. Commun. 2015, 6, 7914 10.1038/ncomms8914. 26228055 PMC4532882

[ref25] Dall’Osto L.; Cazzaniga S.; Zappone D.; Bassi R. Monomeric light harvesting complexes enhance excitation energy transfer from LHCII to PSII and control their lateral spacing in thylakoids. Biochim. Biophys. Acta, Bioenerg. 2020, 1861, 148035 10.1016/j.bbabio.2019.06.007. 31226317

[ref26] Croce R.; Van Amerongen H. Natural strategies for photosynthetic light harvesting. Nat. Chem. Biol. 2014, 10, 492–501. 10.1038/nchembio.1555. 24937067

[ref27] Cleary L.; Chen H.; Chuang C.; Silbey R. J.; Cao J. Optimal fold symmetry of LH2 rings on a photosynthetic membrane. Proc. Natl. Acad. Sci. U.S.A. 2013, 110, 8537–8542. 10.1073/pnas.1218270110. 23650366 PMC3666702

[ref28] Wang D.; Fiebig O. C.; Harris D.; Toporik H.; Ji Y.; Chuang C.; Nairat M.; Tong A. L.; Ogren J. I.; Hart S. M.; Cao J.; Sturgis J. N.; Mazor Y.; Schlau-Cohen G. S. Elucidating Interprotein Energy Transfer Dynamics within the Antenna Network from Purple Bacteria. Proc. Natl. Acad. Sci. U.S.A. 2023, 120, e2220477120 10.1073/pnas.2220477120. 37399405 PMC10334754

[ref29] Ishizaki A.; Fleming G. R. Theoretical examination of quantum coherence in a photosynthetic system at physiological temperature. Proc. Natl. Acad. Sci. U.S.A. 2009, 106, 17255–17260. 10.1073/pnas.0908989106. 19815512 PMC2762676

[ref30] Caruso F.; Chin A. W.; Datta A.; Huelga S. F.; Plenio M. B. Entanglement and entangling power of the dynamics in light-harvesting complexes. Phys. Rev. A 2010, 81, 062346 10.1103/PhysRevA.81.062346.

[ref31] Novoderezhkin V. I.; Palacios M. A.; Van Amerongen H.; Van Grondelle R. Excitation dynamics in the LHCII complex of higher plants: modeling based on the 2.72 Å crystal structure. J. Phys. Chem. B 2005, 109, 10493–10504. 10.1021/jp044082f. 16852271

[ref32] Schlau-Cohen G. S.; Calhoun T. R.; Ginsberg N. S.; Read E. L.; Ballottari M.; Bassi R.; van Grondelle R.; Fleming G. R. Pathways of energy flow in LHCII from two-dimensional electronic spectroscopy. J. Phys. Chem. B 2009, 113, 15352–15363. 10.1021/jp9066586. 19856954

[ref33] Müh F.; Renger T. Refined structure-based simulation of plant light-harvesting complex II: linear optical spectra of trimers and aggregates. Biochim. Biophys. Acta, Bioenerg. 2012, 1817, 1446–1460. 10.1016/j.bbabio.2012.02.016. 22387396

[ref34] Maity S.; Daskalakis V.; Elstner M.; Kleinekathöfer U. Multiscale QM/MM molecular dynamics simulations of the trimeric major light-harvesting complex II. Phys. Chem. Chem. Phys. 2021, 23, 7407–7417. 10.1039/D1CP01011E. 33876100

[ref35] Maity S.; Sarngadharan P.; Daskalakis V.; Kleinekathöfer U. Time-dependent atomistic simulations of the CP29 light-harvesting complex. J. Chem. Phys. 2021, 155, 055103 10.1063/5.0053259. 34364345

[ref36] Do T. N.; Nguyen H. L.; Akhtar P.; Zhong K.; Jansen T. L.; Knoester J.; Caffarri S.; Lambrev P. H.; Tan H.-S. Ultrafast excitation energy transfer dynamics in the LHCII–CP29–CP24 subdomain of plant photosystem II. J. Phys. Chem. Lett. 2022, 13, 4263–4271. 10.1021/acs.jpclett.2c00194. 35522529

[ref37] Shibata Y.; Nishi S.; Kawakami K.; Shen J.-R.; Renger T. Photosystem II does not possess a simple excitation energy funnel: time-resolved fluorescence spectroscopy meets theory. J. Am. Chem. Soc. 2013, 135, 6903–6914. 10.1021/ja312586p. 23537277 PMC3650659

[ref38] Duan H.-G.; Stevens A. L.; Nalbach P.; Thorwart M.; Prokhorenko V. I.; Miller R. D. Two-dimensional electronic spectroscopy of light-harvesting complex II at ambient temperature: a joint experimental and theoretical study. J. Phys. Chem. B 2015, 119, 12017–12027. 10.1021/acs.jpcb.5b05592. 26301382

[ref39] Leng X.; Do T. N.; Akhtar P.; Nguyen H. L.; Lambrev P. H.; Tan H.-S. Hierarchical Equations of Motion Simulation of Temperature-Dependent Two-Dimensional Electronic Spectroscopy of the Chlorophyll a Manifold in LHCII. Chem. - Asian J. 2020, 15, 1996–2004. 10.1002/asia.202000467. 32394636

[ref40] Nguyen H. L.; Do T. N.; Akhtar P.; Jansen T. L.; Knoester J.; Wang W.; Shen J.-R.; Lambrev P. H.; Tan H.-S. An exciton dynamics model of bryopsis corticulans light-harvesting complex II. J. Phys. Chem. B 2021, 125, 1134–1143. 10.1021/acs.jpcb.0c10634. 33478222

[ref41] Agranovich V. M.; Galanin M. D. Electronic Excitation Energy Transfer in Condensed Matter; North-Holland: Amsterdam, 1982.

[ref42] Sinanoğlu O. Modern Quantum Chemistry: Action of Light and Organic Crystals; Academic Press, 1965.

[ref43] Mukai K.; Abe S.; Sumi H. Theory of rapid excitation-energy transfer from B800 to optically-forbidden exciton states of B850 in the antenna system LH2 of photosynthetic purple bacteria. J. Phys. Chem. B 1999, 103, 6096–6102. 10.1021/jp984469g.

[ref44] Jang S.; Newton M. D.; Silbey R. J. Multichromophoric Förster resonance energy transfer. Phys. Rev. Lett. 2004, 92, 218301 10.1103/PhysRevLett.92.218301. 15245322

[ref45] Yang M.; Fleming G. R. Influence of phonons on exciton transfer dynamics: comparison of the Redfield, Förster, and modified Redfield equations. Chem. Phys. 2002, 282, 163–180. 10.1016/S0301-0104(02)00604-3.

[ref46] Hwang-Fu Y.-H.; Chen W.; Cheng Y.-C. A coherent modified Redfield theory for excitation energy transfer in molecular aggregates. Chem. Phys. 2015, 447, 46–53. 10.1016/j.chemphys.2014.11.026.

[ref47] Kreisbeck C.; Kramer T.; Aspuru-Guzik A. Scalable High-Performance Algorithm for the Simulation of Exciton Dynamics. Application to the Light-Harvesting Complex II in the Presence of Resonant Vibrational Modes. J. Chem. Theory Comput. 2014, 10, 4045–4054. 10.1021/ct500629s. 26588548

[ref48] Varvelo L.; Lynd J. K.; Bennett D. I. G. Formally Exact Simulations of Mesoscale Exciton Dynamics in Molecular Materials. Chem. Sci. 2021, 12, 9704–9711. 10.1039/D1SC01448J. 34349941 PMC8293828

[ref49] See: https://github.com/GHlacour/NISE_2017 for NISE_2017 (Accessed February 2, 2022).

[ref50] Sardjan A. S.; Westerman F. P.; Ogilvie J. P.; Jansen T. L. C. Observation of Ultrafast Coherence Transfer and Degenerate States with Polarization-Controlled Two-Dimensional Electronic Spectroscopy. J. Phys. Chem. B 2020, 124, 9420–9427. 10.1021/acs.jpcb.0c08126. 32990439 PMC7586392

[ref51] Jansen T. L. C.; Knoester J. Nonadiabatic Effects in the Two-Dimensional Infrared Spectra of Peptides: Application to Alanine Dipeptide. J. Phys. Chem. B 2006, 110, 22910–22916. 10.1021/jp064795t. 17092043

[ref52] Liang C.; Jansen T. L. C. An Efficient N ^3^ -Scaling Propagation Scheme for Simulating Two-Dimensional Infrared and Visible Spectra. J. Chem. Theory Comput. 2012, 8, 1706–1713. 10.1021/ct300045c. 26593664

[ref53] Kunsel T.; Tiwari V.; Matutes Y. A.; Gardiner A. T.; Cogdell R. J.; Ogilvie J. P.; Jansen T. L. C. Simulating Fluorescence-Detected Two-Dimensional Electronic Spectroscopy of Multichromophoric Systems. J. Phys. Chem. B 2019, 123, 394–406. 10.1021/acs.jpcb.8b10176. 30543283 PMC6345114

[ref54] van Hengel C. D. N.; van Adrichem K. E.; Jansen T. L. C. Simulation of Two-Dimensional Infrared Raman Spectroscopy with Application to Proteins. J. Chem. Phys. 2023, 158, 064106 10.1063/5.0138958. 36792507

[ref55] Jansen T. L. C.; Auer B. M.; Yang M.; Skinner J. L. Two-Dimensional Infrared Spectroscopy and Ultrafast Anisotropy Decay of Water. J. Chem. Phys. 2010, 132, 224503 10.1063/1.3454733. 20550404

[ref56] Bondarenko A. S.; Knoester J.; Jansen T. L. Comparison of methods to study excitation energy transfer in molecular multichromophoric systems. Chem. Phys. 2020, 529, 110478 10.1016/j.chemphys.2019.110478.

[ref57] Müh F.; Madjet M. E.-A.; Adolphs J.; Abdurahman A.; Rabenstein B.; Ishikita H.; Knapp E.-W.; Renger T. α-Helices direct excitation energy flow in the Fenna–Matthews–Olson protein. Proc. Natl. Acad. Sci. U.S.A. 2007, 104, 16862–16867. 10.1073/pnas.0708222104. 17940020 PMC2040394

[ref58] Olbrich C.; Kleinekathöfer U. Time-Dependent Atomistic View on the Electronic Relaxation in Light-Harvesting System II. J. Phys. Chem. B 2010, 114, 12427–12437. 10.1021/jp106542v. 20809619

[ref59] Ma J.; Cao J. Förster Resonance Energy Transfer, Absorption and Emission Spectra in Multichromophoric Systems. I. Full Cumulant Expansions and System-Bath Entanglement. J. Chem. Phys. 2015, 142, 094106 10.1063/1.4908599. 25747060

[ref60] Wu J.; Cao J. Higher-Order Kinetic Expansion of Quantum Dissipative Dynamics: Mapping Quantum Networks to Kinetic Networks. J. Chem. Phys. 2013, 139, 044102 10.1063/1.4812781. 23901955

[ref61] Wu J.; Gong Z.; Tang Z. Generalized Quantum Kinetic Expansion: Higher-order Corrections to Multichromophoric Förster Theory. J. Chem. Phys. 2015, 143, 074102 10.1063/1.4928634. 26298110

[ref62] Mukai K.; Abe S.; Sumi H. Theory of Rapid Excitation-Energy Transfer from B800 to Optically-Forbidden Exciton States of B850 in the Antenna System LH2 of Photosynthetic Purple Bacteria. J. Phys. Chem. B 1999, 103, 6096–6102. 10.1021/jp984469g.

[ref63] Jang S. Generalization of the Förster Resonance Energy Transfer Theory for Quantum Mechanical Modulation of the Donor-Acceptor Coupling. J. Chem. Phys. 2007, 127, 174710 10.1063/1.2779031. 17994845

[ref64] Bader J. S.; Berne B. J. Quantum and Classical Relaxation Rates from Classical Simulations. J. Chem. Phys. 1994, 100, 8359–8366. 10.1063/1.466780.

[ref65] Oxtoby D. W. Vibrational Relaxation in Liquids. Annu. Rev. Phys. Chem. 1981, 32, 77–101. 10.1146/annurev.pc.32.100181.000453.

[ref66] Egorov S. A.; Everitt K. F.; Skinner J. L. Quantum Dynamics and Vibrational Relaxation. J. Phys. Chem. A 1999, 103, 9494–9499. 10.1021/jp9919314.

[ref67] Bastida A.; Cruz C.; Zuniga J.; Requena A.; Miguel D. A Modified Ehrenfest Method That Achieves Boltzmann Quantum State Populations. Chem. Phys. Lett. 2006, 417, 53–57. 10.1016/j.cplett.2005.10.008.

[ref68] Aghtar M.; Liebers J.; Strümpfer J.; Schulten K.; Kleinekathöfer U. Juxtaposing Density Matrix and Classical Path-Based Wave Packet Dynamics. J. Chem. Phys. 2012, 136, 214101 10.1063/1.4723669. 22697524 PMC3376871

[ref69] Saraceno P.; Sláma V.; Cupellini L. First-Principles Simulation of Excitation Energy Transfer and Transient Absorption Spectroscopy in the CP29 Light-Harvesting Complex. J. Chem. Phys. 2023, 159, 184112 10.1063/5.0170295. 37962444

[ref70] Chuang C.; Knoester J.; Cao J. Scaling Relations and Optimization of Excitonic Energy Transfer Rates between One-Dimensional Molecular Aggregates. J. Phys. Chem. B 2014, 118, 7827–7834. 10.1021/jp4124502. 24645980

[ref71] Lloyd S.; Mohseni M. Symmetry-Enhanced Supertransfer of Delocalized Quantum States. New J. Phys. 2010, 12, 075020 10.1088/1367-2630/12/7/075020.

[ref72] Pugžlys A.; Augulis R.; van Loosdrecht P. H. M.; Didraga C.; Malyshev V. A.; Knoester J. Temperature-Dependent Relaxation of Excitons in Tubular Molecular Aggregates: Fluorescence Decay and Stokes Shift. J. Phys. Chem. B 2006, 110, 20268–20276. 10.1021/jp062983d. 17034206

[ref73] Bondarenko A. S.; Patmanidis I.; Alessandri R.; Souza P. C. T.; Jansen T. L. C.; de Vries A. H.; Marrink S. J.; Knoester J. Multiscale Modeling of Molecular Structure and Optical Properties of Complex Supramolecular Aggregates. Chem. Sci. 2020, 11, 11514–11524. 10.1039/D0SC03110K. 34094396 PMC8162738

[ref74] Thouless D. J. Electrons in Disordered Systems and the Theory of Localization. Phys. Rep. 1974, 13, 93–142. 10.1016/0370-1573(74)90029-5.

[ref75] Chuang C.; Bennett D. I.; Caram J. R.; Aspuru-Guzik A.; Bawendi M. G.; Cao J. Generalized Kasha’s Model: T-Dependent Spectroscopy Reveals Short-Range Structures of 2D Excitonic Systems. Chem 2019, 5, 3135–3150. 10.1016/j.chempr.2019.08.013.

[ref76] Blankenship R. E. Molecular Mechanisms of Photosynthesis, 3rd ed.; Wiley: Oxfort, U.K., 2021.

[ref77] Varvelo L.; Lynd J. K.; Citty B.; Kühn O.; Raccah D. I. G. B. Formally Exact Simulations of Mesoscale Exciton Diffusion in a Light-Harvesting 2 Antenna Nanoarray. J. Phys. Chem. Lett. 2023, 14, 3077–3083. 10.1021/acs.jpclett.3c00086. 36947483 PMC10069740

[ref78] Şener M.; Strümpfer J.; Timney J. A.; Freiberg A.; Hunter C. N.; Schulten K. Photosynthetic Vesicle Architecture and Constraints on Efficient Energy Harvesting. Biophys. J. 2010, 99, 67–75. 10.1016/j.bpj.2010.04.013. 20655834 PMC2895385

[ref79] Cleary L.; Chen H.; Chuang C.; Silbey R. J.; Cao J. Optimal Fold Symmetry of LH2 Rings on a Photosynthetic Membrane. Proc. Natl. Acad. Sci. U.S.A. 2013, 110, 8537–8542. 10.1073/pnas.1218270110. 23650366 PMC3666702

[ref80] Humphrey W.; Dalke A.; Schulten K. VMD - Visual Molecular Dynamics. J. Mol. Graphics 1996, 14, 33–38. 10.1016/0263-7855(96)00018-5. 8744570

[ref81] Papiz M. Z.; Prince S. M.; Howard T.; Cogdell R. J.; Isaacs N. W. The Structure and Thermal Motion of the B800–850 LH2 Complex from Rps.Acidophila at 2.0Å Resolution and 100K: New Structural Features and Functionally Relevant Motions. J. Mol. Biol. 2003, 326, 1523–1538. 10.1016/S0022-2836(03)00024-X. 12595263

[ref82] Prince S.; Papiz M.; Freer A.; McDermott G.; Hawthornthwaite-Lawless A.; Cogdell R.; Isaacs N. Apoprotein structure in the LH2 complex from Rhodopseudomonas acidophila strain 10050: modular assembly and protein pigment interactions. J. Mol. Biol. 1997, 268, 412–423. 10.1006/jmbi.1997.0966. 9159480

[ref83] Madjet M. E.; Abdurahman A.; Renger T. Intermolecular Coulomb Couplings from Ab Initio Electrostatic Potentials: Application to Optical Transitions of Strongly Coupled Pigments in Photosynthetic Antennae and Reaction Centers. J. Phys. Chem. B 2006, 110, 17268–17281. 10.1021/jp0615398. 16928026

[ref84] Renger T. Theory of Excitation Energy Transfer: From Structure to Function. Photosynth. Res. 2009, 102, 471–485. 10.1007/s11120-009-9472-9. 19653118

[ref85] van der Vegte C. P.; Prajapati J. D.; Kleinekathöfer U.; Knoester J.; Jansen T. L. C. Atomistic Modeling of Two-Dimensional Electronic Spectra and Excited-State Dynamics for a Light Harvesting 2 Complex. J. Phys. Chem. B 2015, 119, 1302–1313. 10.1021/jp509247p. 25554919

[ref86] Kennis J. T. M.; Streltsov A. M.; Vulto S. I. E.; Aartsma T. J.; Nozawa T.; Amesz J. Femtosecond Dynamics in Isolated LH2 Complexes of Various Species of Purple Bacteria. J. Phys. Chem. B 1997, 101, 7827–7834. 10.1021/jp963359b.

[ref87] Buckingham A. D. Molecular quadrupole moments. Chem. Soc. Rev. 1959, 13, 183–214. 10.1039/qr9591300183.

[ref88] Maineult W.; Pelle B.; Faoro R.; Arimondo E.; Pillet P.; Cheinet P. Dipole–quadrupole Förster resonance in cesium Rydberg gas. J. Phys. B:At., Mol. Opt. Phys. 2016, 49, 214001 10.1088/0953-4075/49/21/214001.

[ref89] Jackson J. D. Classical Electrodynamics; John Wiley & Sons, 2021.

[ref90] Stone A. J. The Theory of Intermolecular Forces; International Series of Monographs on Chemistry; Oxford University Press: Oxford, 1996; Vol. 32.

[ref91] Jang S. J. Robust and Fragile Quantum Effects in the Transfer Kinetics of Delocalized Excitons between B850 Units of LH2 Complexes. J. Phys. Chem. Lett. 2018, 9, 6576–6583. 10.1021/acs.jpclett.8b02641. 30383380

[ref92] Jang S.; Rivera E.; Montemayor D. Molecular Level Design Principle behind Optimal Sizes of Photosynthetic LH2 Complex: Taming Disorder through Cooperation of Hydrogen Bonding and Quantum Delocalization. J. Phys. Chem. Lett. 2015, 6, 928–934. 10.1021/acs.jpclett.5b00078. 26262847

[ref93] Jang S.; Hoyer S.; Fleming G.; Whaley K. B. Generalized Master Equation with Non-Markovian Multichromophoric Förster Resonance Energy Transfer for Modular Exciton Densities. Phys. Rev. Lett. 2014, 113, 188102 10.1103/PhysRevLett.113.188102. 25396397

[ref94] Tempelaar R.; Jansen T. L. C.; Knoester J. Vibrational Beatings Conceal Evidence of Electronic Coherence in the FMO Light-Harvesting Complex. J. Phys. Chem. B 2014, 118, 12865–12872. 10.1021/jp510074q. 25321492

[ref95] Thyrhaug E.; Tempelaar R.; Alcocer M. J.; Žídek K.; Bína D.; Knoester J.; Jansen T. L.; Zigmantas D. Identification and characterization of diverse coherences in the Fenna–Matthews–Olson complex. Nat. Chem. 2018, 10, 780–786. 10.1038/s41557-018-0060-5. 29785033

[ref96] Klinger A.; Lindorfer D.; Müh F.; Renger T. Normal Mode Analysis of Spectral Density of FMO Trimers: Intra- and Intermonomer Energy Transfer. J. Chem. Phys. 2020, 153, 215103 10.1063/5.0027994. 33291900

[ref97] Thyrhaug E.; Zidek K.; Dostal J.; Bina D.; Zigmantas D. Exciton Structure and Energy Transfer in the Fenna-Matthews- Olson Complex. J. Phys. Chem. Lett. 2016, 7, 1653–1660. 10.1021/acs.jpclett.6b00534. 27082631

[ref98] Vulto S. I. E.; de Baat M. A.; Neerken S.; Nowak F. R.; van Amerongen H.; Amesz J.; Aartsma T. J. Excited State Dynamics in FMO Antenna Complexes from Photosynthetic Green Sulfur Bacteria: A Kinetic Model. J. Phys. Chem. B 1999, 103, 8153–8161. 10.1021/jp984702a.

[ref99] Zhong K.; Nguyen H. L.; Do T. N.; Tan H.-S.; Knoester J.; Jansen T. L. Coarse-Grained Approach to Simulate Signatures of Excitation Energy Transfer in Two-Dimensional Electronic Spectroscopy of Large Molecular Systems. J. Chem. Theory Comput. 2024, 20, 6111–6124. 10.1021/acs.jctc.4c00413. 38996082 PMC11270824

[ref100] LAPACK https://www.netlib.org/lapack/.

[ref101] See: https://github.com/GHlacour/NISE_tutorials for NISE_Tutorials (Accessed Oct 1, 2024).

